# Nucleation processes of nanobubbles at a solid/water interface

**DOI:** 10.1038/srep24651

**Published:** 2016-04-19

**Authors:** Chung-Kai Fang, Hsien-Chen Ko, Chih-Wen Yang, Yi-Hsien Lu, Ing-Shouh Hwang

**Affiliations:** 1 Institute of Physics, Academia Sinica, Nankang, Taipei 115, Taiwan

## Abstract

Experimental investigations of hydrophobic/water interfaces often return controversial results, possibly due to the unknown role of gas accumulation at the interfaces. Here, during advanced atomic force microscopy of the initial evolution of gas-containing structures at a highly ordered pyrolytic graphite/water interface, a fluid phase first appeared as a circular wetting layer ~0.3 nm in thickness and was later transformed into a cap-shaped nanostructure (an interfacial nanobubble). Two-dimensional ordered domains were nucleated and grew over time outside or at the perimeter of the fluid regions, eventually confining growth of the fluid regions to the vertical direction. We determined that interfacial nanobubbles and fluid layers have very similar mechanical properties, suggesting low interfacial tension with water and a liquid-like nature, explaining their high stability and their roles in boundary slip and bubble nucleation. These ordered domains may be the interfacial hydrophilic gas hydrates and/or the long-sought chemical surface heterogeneities responsible for contact line pinning and contact angle hysteresis. The gradual nucleation and growth of hydrophilic ordered domains renders the original homogeneous hydrophobic/water interface more heterogeneous over time, which would have great consequence for interfacial properties that affect diverse phenomena, including interactions in water, chemical reactions, and the self-assembly and function of biological molecules.

Gases dissolved in water tend to accumulate at the interfaces between hydrophobic solids and water to form cap-shaped structures that are nanometers in height; these structures are known as interfacial nanobubbles (INBs) or surface nanobubbles^1^,^2^,^3^,^4^,^5^,^6^,^7^,^8^,^9^,^10^. INBs have attracted much attention because of their potential implications for various interfacial phenomena and technical applications, such as long-range attractive forces between hydrophobic surfaces in solutions^11^, liquid slippage at hydrophobic walls^12^,^13^,^14^, the stability of colloidal systems^15^, and bio-molecular adsorption^16^. INBs are also proposed to be the gas micronuclei that are responsible for bubble formation at solid/water interfaces^17^. However, the nature of INBs remains unknown, and the mechanisms responsible for the above phenomena are not clear.

To date, most studies have focused on why INBs exhibit high stability and why they adopt a rather flat morphology. The lifetime of an INB should be much less than 1 ms based on classical diffusion theory^18^,^19^, but experimental observations have indicated that INBs can persist for days, which is at least 10 orders of magnitude longer than the theoretical prediction^5^,^6^. Although several models have been proposed to explain this unexpected stability, no consensus has been reached. A very recent model was based on the pinning of the three-phase INB-water-surface contact line^20^,^21^, which was attributed to omnipresent chemical and geometrical surface heterogeneities^21^ of unknown origin. Such surface heterogeneities were also suggested to lead to contact angle hysteresis, the difference between an advancing and a receding contact angle, for water droplets on solid surfaces^22^,^23^,^24^.

Another fundamental but rarely addressed issue is the mechanism by which INBs nucleate at hydrophobic/water interfaces. Here, we used advanced atomic force microscopy (AFM) to investigate the initial formation of gas-containing structures at the interface between water and a mildly hydrophobic solid, highly ordered pyrolytic graphite (HOPG). Our observations, which were conducted in the frequency-modulation (FM) and PeakForce (PF) modes, provide important insights into the behaviours of dissolved gases in water and at hydrophobic/water interfaces, including formation of INBs, their nature, their high stability, the pinning of the three-phase contact lines, boundary slip, and bubble nucleation. This new understanding highlights new directions for further quantitative investigation of these behaviours under various conditions and at various interfaces, which will allow accurate prediction and control of these behaviours. These advances will strongly impact research and technology in diverse fields, such as physics, chemistry, biology, and medicine.

## Results

### Formation of gas-containing structures at a HOPG/water interface

**Figure 1:**
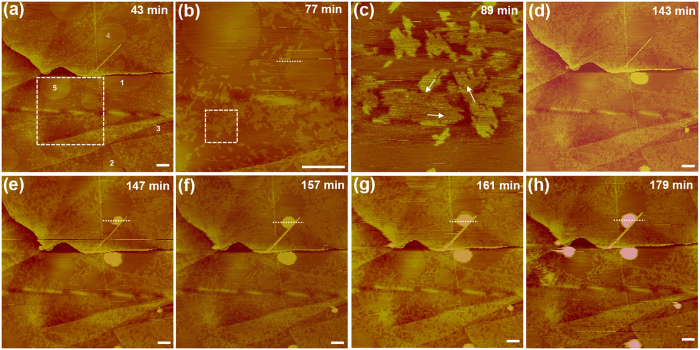
Topographic images of the formation of gas-containing structures at a HOPG/water interface acquired with FM-AFM. Time points are relative to the time of water deposition (t* *= 0). Scale bar, 400 nm. Numbered regions in (**a**) exhibit a circular, thin layer covering the HOPG substrate that changed in morphology over time. The numbers in panel (**a**) are not shown in the other panels. (**b**) A higher-resolution image acquired inside the dashed box in (**a**) at a later time. The height profile measured along a white dashed line is shown in Supplementary Fig. S1a. (**c**) A high-resolution image of bright speckles (inside the dashed box in (**b**) at a later time), consisting of domains of a row-like pattern with three orientations (arrows) that are parallel to the orientations of the HOPG substrate. The domains with approximately horizontal row orientation (the fast scan direction) cannot be clearly resolved. A cap-shaped structure appeared in Region 4 in (**e**) and exhibited growth in both the vertical and lateral dimensions (**f–h**). The height profiles along a white dashed line across the INB from (**e–h**) are depicted in Supplementary Fig. S1b.

Figure 1 shows an example of our observations after chilled water (see Methods) was deposited onto a freshly cleaved HOPG surface. Several regions, several of which are numbered in Fig. 1a, exhibited a thin circular layer covering the HOPG substrate. Notice that several circular layers had a diameter as large as ~1 μm, and some had edges bounded by defects such as substrate step edges. The height of the circular layer was 0.3–0.4 nm (Supplementary Fig. S1a). In addition, bright speckles were scattered at the interface outside the circular layered regions (Fig. 1a). Higher-resolution images showed that the speckles were small domains with an ordered row-like structure (Fig. 1b,c) that increased in coverage of the interface over time (Fig. 1d–h). This structure and its nucleation and growth behaviours resemble the ordered pattern reported in our earlier studies of the HOPG/water interface using pre-degassed water under air or nitrogen^25^,^26^,^27^,^28^.

At *t *= 143 min, the thin layers in regions 1–3 transformed into a cap-shaped INB (Fig. 1d). Four minutes later, a small cap-shaped protrusion appeared on the thin layer in region 4 (Fig. 1e). The thin layer gradually receded, revealing its fluid nature, while the protrusion grew in both the lateral and vertical dimensions (Fig. 1e–h and Supplementary Fig. S1b). The ordered domains covered more of the interface surface over time and confined the lateral growth of fluid layers at late stages. After the edges of the fluid layer receded, a region of bare HOPG surface bounded by ordered domains and substrate step edges was exposed (Fig. 1e–h), indicating that no ordered structure was formed inside the fluid layer. In the area near the fluid layer in region 4, several smaller fluid layers displayed a circular shape at *t* = 43 min but grew laterally until their boundaries were confined by the ordered domains (Supplementary Fig. S2).

Another AFM observation revealed two thin circular layers >1 μm in diameter; both displayed a very rapid (<1 s) transition from a fluid layer to a cap-shaped INB (Supplementary Fig. S3). Similar to the observation in Fig. 1, a large, bare HOPG surface was exposed directly after the transition, but small ordered domains gradually nucleated inside the bare HOPG regions (Supplementary Fig. S3e), and the two INBs grew in the vertical and lateral directions (Supplementary Fig. S3g,h). We note that the ordered domains exhibited a smaller dissipation than the bare HOPG surface (Supplementary Figs S2 and S3), consistent with our previous observations^25^,^26^,^27^,^28^,^29^.

**Figure 2:**
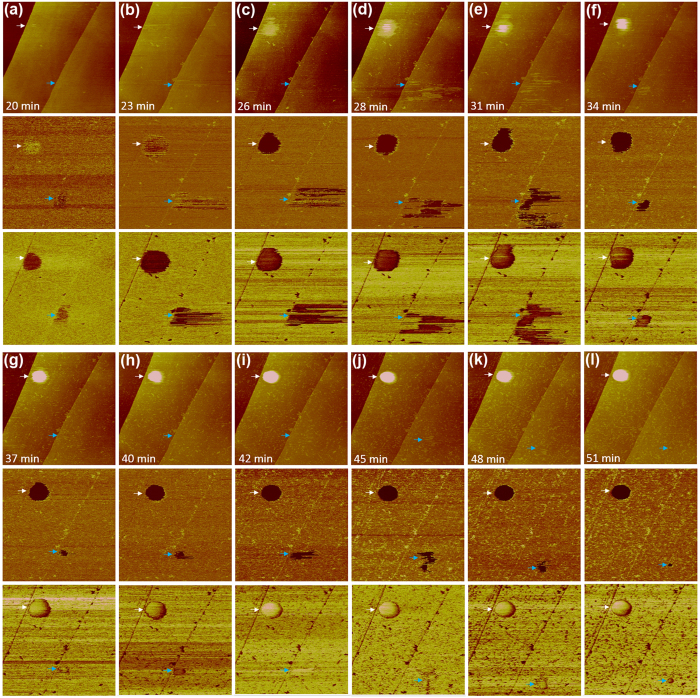
AFM images (PF mode, 250 pN) of the transition from a 2D fluid layer to a 3D structure. The scan area is 2 μm × 2 μm. Chilled water was rapidly heated to 45 °C and deposited on a freshly cleaved HOPG substrate. Images were acquired continuously with a scanning rate of ~3 min per image. The top, middle, and bottom rows of each panel depict the topographic, stiffness (Young’s modulus), and adhesion maps, respectively. Two fluid regions are highlighted with white and blue arrows.

We detected a different transformation process for smaller fluid regions when chilled water was rapidly heated to 45 °C before deposition (Fig. 2). At *t *= 20 min and *t *= 23 min, gas adsorption at the interface was evident in the stiffness and adsorption maps, but was barely detectable in the topographic images acquired in PF mode (white arrow, Fig. 2a,b). This difference occurred because the AFM tip usually penetrates into fluid structures to a certain depth that depends on the hydrophobicity of the tip^30^, in order to achieve a positive pre-set peak force due to the capillary force between the tip and the fluid structure^29^. When the fluid structure is too thin, the tip traces the profile of the underlying stiff structure (the substrate, in this case). At *t *= 26 min, the topographic image began to reveal the presence of a circular layer 0.4–0.5 nm in thickness (white arrow, Fig. 2c). At *t *= 28 min, the apparent height of the fluid layer continued to grow, and a small protrusion appeared near the centre of the layer (Fig. 2d). A cap-shaped INB eventually formed (Fig. 2f–l).

The stiffness maps (middle rows in Fig. 2) indicate that the fluid phase is softer than the other regions of the interface; thus, the stiffness maps enable easy detection of the fluid regions. However, the quantitative stiffness value may be not accurately extracted for a structure <2 nm in thickness, a limitation that occasionally led to a reversal of the stiffness contrast of the fluid structures (Fig. 2a). Adhesion contrast between the fluid regions and bare HOPG regions varies from tip to tip, but still allows distinction between these two regions. The stiffness and adhesion maps did not provide enough contrast to distinguish between the fluid layer and the cap-shaped structure (Fig. 2d,e), suggesting that these two structures share a very similar nature. The ordered domains are visible in Fig. 2, but they appear as white and dark speckles in the topographic and adhesion maps, respectively, due to the large scan area of the images. The ordered domains consistently exhibited a higher stiffness than the fluid structures (Fig. 2). However, they appeared slightly stiffer or softer than the bare HOPG regions, depending on the tip and operating conditions, again because the ordered domains were <2 nm in thickness and their stiffness values could not be accurately extracted.

**Figure 3:**
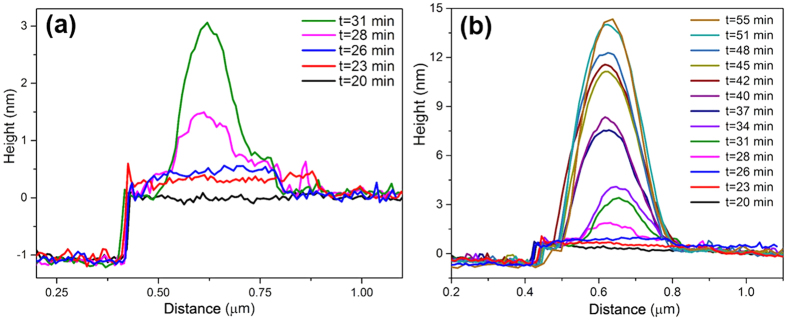
Apparent height profiles of the fluid structure indicated by a white arrow in Fig. 2. (**a**) Profiles acquired from *t *= 20 min to *t *= 31 min, during the initial stages of INB formation. (**b**) Overall profiles acquired from *t *= 20 min to *t *= 55 min.

The apparent height of the fluid region highlighted with a white arrow in Fig. 2 gradually increased over time (Fig. 3a). The real thickness of the fluid structures (the thin layer and the cap-shaped structure) should be larger than the apparent height due to the effect of tip-penetration depth; thus, the fluid layer in Fig. 2c,d is more than one molecule thick. After a protrusion appeared at *t *= 28 min, both the lateral size and the apparent height of the protrusion continued to grow until *t *= 37 min, after which growth mainly occurred in the vertical dimension (the lateral size remained roughly the same) (Fig. 3b). The growth behaviour of INBs after *t *= 37 min resembled that reported by Zhang *et al*. (who changed the air-supersaturation level), which Zhang *et al*. attributed to the pinning of the three-phase contact line^31^. The apparent height of the fluid region indicated with a white arrow in Fig. 2 continued to increase over time (Supplementary Fig. S4); the lateral area initially increased, but gradually decreased at later times (Supplementary Fig. S4), in contrast to the rapid reduction in the lateral size of the fluid layer evident in Fig. 1 and Supplementary Fig. S3.

Another region of gas adsorption is visible in the adhesion and stiffness maps in Fig. 2 (blue arrow). The lateral shape of this region was irregular and changed from frame to frame (Fig. 2), suggesting that the gas structure was highly mobile in this region during tip scanning. This mobility appeared to slow at *t *= 48 min (Fig. 2k), and the structure transformed into a small INB at *t *= 51 min (Fig. 2l). The high mobility of this gas structure seems to be related to its small size; this gas structure is barely detectable in the topographic images in Fig. 2.

Enlarging the scan area of Fig. 2 revealed additional INBs (Supplementary Fig. S5a), indicating that the 2D-to-3D transition of fluid regions is spontaneous. However, we cannot rule out the possibility that the scanning tip may facilitate this transition. Ordered domains of the row-like structure were evident in regions outside the INBs (Supplementary Fig. S5b,c). Force curve measured on the INB indicated with a white arrow in Fig. 2 clearly shows a snap-in when the tip touches the INB (Supplementary Fig. S5d).

**Figure 4:**
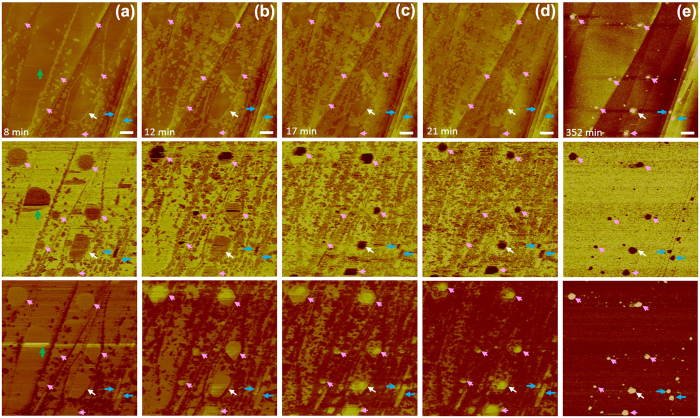
PF (220 pN) images of gas-containing structures at a HOPG/water interface after the deposition of chilled water. Top, middle, and bottom rows contain height, stiffness, and adhesion maps, respectively. Scale bar, 500 nm. The green arrow in (**a**) highlights a fluid layer that disappeared at a later time (**b**). The white, blue, and pink arrows indicate fluid regions that later transformed into a cap-shaped structure (**e**). Two blue arrows indicate fluid regions that were confined by two step edges, yielding rectangular gas structures (**a**–**d**). Note that the ordered domains exhibited less adhesion than the bare HOPG surface and the fluid structures. In (**e**), the entire interface (except for the INBs) was dark on the adhesion maps (bottom row) and bright on the stiffness maps (middle row), indicating that the interface was nearly completely covered by the ordered domains. The regions of the fluid structures can be distinguished easily in the stiffness maps because they exhibited less stiffness than the ordered domains and HOPG substrate. This AFM tip was relatively hydrophobic, yielding a penetration depth for the fluid regions that was larger than that obtained with the tips used to acquire the data in the other figures presented in this work. A cap-shaped structure is visible in a topographic image only when its height is larger than the tip penetration depth. In addition, it is difficult to distinguish between INBs and fluid layers from the stiffness and adhesion maps. Thus, the INBs may have formed well before we detected the cap-shaped structures in the topographic (height) maps.

We identified an interesting case of relatively fast nucleation and growth of the ordered domains (Fig. 4). Fluid layers (some indicated with coloured arrows in Fig. 4a) and low-coverage ordered domains appeared in the stiffness and adhesion maps at *t* = 8 min (Fig. 4a). Confinement of the fluid regions occurred at *t* = 12 min (Fig. 4b). The fluid regions seemed to be present at a lower height than the surrounding ordered domains and were barely distinguishable from the bare HOPG regions in the topographic images (Fig. 4a,b). Portions of the outer parts of the fluid regions transformed into ordered domains, decreasing the lateral size of the fluid regions (Fig. 4b–d). Cap-shaped protrusions were evident in the topographic images at *t*~50 min (data not shown). At *t* = 352 min, all regions highlighted by coloured arrows in Fig. 4 transformed into cap-shaped structures (Fig. 4e), with the exception of the region denoted by a green arrow in Fig. 4a.

**Figure 5:**
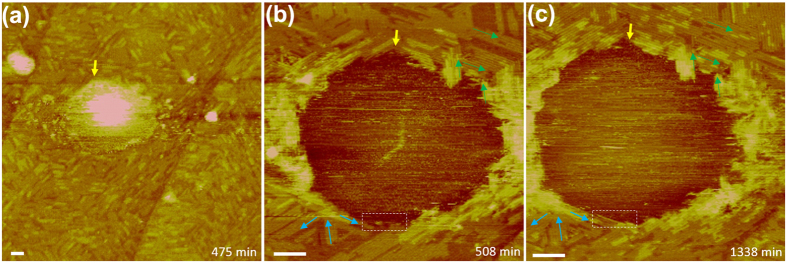
High-resolution topographic images (PF mode) around the region indicated with a white arrow in Fig. 4 acquired at later times. The peak force was set to 220 pN for (**a**) and 660 pN for (**b,c**). The yellow arrow serves as a marker for comparison among the three panels. Scale bar, 70 nm. The images in (**b,c**) were acquired with the fast scan direction rotated 20° clockwise relative to that in (**a**); the row-like patterns could not be well resolved when the row orientation was parallel with the fast scan direction. Blue and green arrows are indicated in (**b**,**c**) for comparison of structural changes over time. Growth of more layers of ordered structures at the outer edges of the INB is evident in (**c**). The region outlined by a white dashed box in (**b,c**) highlights the growth of the ordered structures at a boundary of the INB.

We acquired a higher-resolution topographic image around the cap-shaped structure indicated with a white arrow in Fig. 4 at *t* = 475 min (Fig. 5a). At this time point, the INB was already surrounded by several layers of ordered structures. When we scanned the INB at a higher peak force, the INB appeared as a depression surrounded by multi-layer ordered domains (Fig. 5b). We previously demonstrated that a higher peak force results in a lower surface profile of an INB because the tip penetrates more deeply into the fluid structure^29^. Figure 5b shows that only bare HOPG substrate, with no ordered structures, was present under the INB. The edges of the surrounding ordered structures were faceted and roughly hexagonal (Fig. 5b). The thickness of the ordered structures was larger at the perimeter of the INB than at other locations away from the INB, suggesting that the formation of ordered structures is more favourable next to a fluid structure. Images acquired at later times at this high peak force revealed the growth of the ordered structures at the outer boundaries of the INB (Fig. 5c) and clearly visualised strong confinement of the INB by the surrounding ordered structures.

### Nature of the fluid layers and INBs at the HOPG/water interface

The surprising formation of a large wetting layer (1 μm in diameter) one molecule in thickness (Fig. 1 and Supplementary Fig. S3) indicates the presence of an adsorbed gas film that is in a liquid-like state rather than in a vapour phase. Most importantly, this observation revealed that there is a tendency for the adsorbed gas structure to spread as much as possible along the HOPG/water interface (Supplementary Note 1). This excellent wetting and spreading behaviour indicates that the interfacial tension (interface energy per unit area) between the adsorbed gas film and water, **σ**_**film-water**_, should be considerably smaller than the surface tension of water **(σ**_**vapour-water**_* *= 72 mN/m) (Supplementary Note 2). The high surface tension of water is the driving force that retains semispherical gas bubbles microns in size or larger at solid/water interfaces.

Based on several observations, we conclude that INBs are mainly liquid-like gas condensates at the solid/water interface, rather than surface bubbles with low-density gases in their vapour phase. First, we observed a relatively continuous height increase during the 2D-to-3D transition of the fluid structure (Figs 2 and 3), without an abrupt and large volume change corresponding to a liquid-vapour phase transition. Second, our PF measurements (Figs 2 and 4) indicate that the mechanical properties (stiffness and adhesion) of the fluid layers and the cap-shaped structures are nearly indistinguishable. Third, we determined that a cap-shaped structure can form on top of a fluid layer and persist for more than 10 min (Figs 1 and 2), demonstrating that these two structures have similar chemical potentials and a similar nature. Since INBs exhibit good wetting with the interface with a very small contact angle (5°~25° measured from the gas side^5^,^6^,^7^,^8^), we expect that the interfacial tension between an INB and water is significantly smaller than the surface tension of water.

The low interfacial tension of INBs may account for the surprisingly high stability of INBs observed experimentally. It also solves two mysteries in the science of solid/water interfaces: boundary slip and the nucleation of gas bubbles in water. An INB may serve as a highly deformable liquid-like layer of low viscosity that reduces the drag of flowing water over a hydrophobic wall; this function would explain the mechanism of the boundary-slip effect proposed by Vinogradova^32^, who also proposed that gas bubbles with high surface tension would not deform easily and thus could not contribute to drag reduction^33^. Consistent with this hypothesis, gas bubbles trapped at solid/water interfaces acted as anti-lubricants and increased drag^34^. INBs were suggested to be responsible for boundary slip on hydrophobic surfaces, but they were generally considered to be air bubbles on the nanometre scale^13^,^14^. Our demonstration of the drastically different natures of INBs and gas bubbles resolves this contradiction. The low interfacial tension of INBs may also underlie the nucleation of gas bubbles in water, and thus large INBs may be the long-sought micronuclei for gas bubble formation in water (Supplementary Note 3). A recent work by Attard reported surface tensions of INBs substantially lower than that of water^35^. Quantitative measurement of the interfacial tension between an INB and water, which was beyond the scope of the present investigation, will enable further interrogation of these hypotheses.

The high surface tension of water makes small gas bubbles energetically unfavourable, because the interfacial energy contributes to a larger portion of the total energy (bulk energy plus interfacial energy) as the size of a gas bubble decreases. Gas bubbles microns in size or larger exhibit such a trend, but INBs do not—INBs are extremely stable. We suspect that the high surface tension of water may be the thermodynamic driving force that condenses very small gas bubbles into gas condensates, which become energetically favourable due to a much lower interfacial tension with water and a smaller interfacial area^26^,^27^,^29^. As described above, the low interfacial tension of INBs with water solves several outstanding mysteries, including boundary slip and the nucleation of gas bubbles. The low interfacial tension is also consistent with several recent experimental results. First, transmission electron microscopy of merging INBs^36^ showed that INBs are highly deformable and can exhibit a shape that strongly deviates from a semisphere, e.g. a dumbbell shape, for tens of seconds, which is considerably longer than the merging process for surface bubbles with a diameter of tens of microns to centimetres (<1 s). Second, INBs did not burst when a macroscopic surface bubble (containing water vapour) grew and moved across the INBs^37^; instead, a water film wet the INBs and nucleated a growing water droplet through vapour condensation. This process reflects the favourable affinity of INBs for water, which is consistent with the low interfacial tension proposed here but not with the high interfacial tension associated with a gas bubble in water. Third, giant INBs with a lateral diameter of 8–10 μm were reported in recent years^38^,^39^. They exhibited a rather flat and stable morphology similar to that of smaller INBs^38^. Surface gas bubbles with this lateral diameter are visible on typical optical microscopy and exhibit a semispherical shape with a much higher height/diameter ratio than INBs. In addition, these small gas bubbles are unstable and grow or shrink within a few seconds, depending on the dissolved gas concentration. In contrast, the morphology of INBs changes only slightly many hours after the change in gas concentration^31^, indicating very inefficient gas transfer between INBs and the surrounding water. The observations of giant INBs indicate that it is their nature, rather than their size, that differentiates INBs from typical surface gas bubbles. Thus, we suggest that “nanobubble” is not an appropriate name for these cap-shaped fluid structures that consist of liquid-like gas condensates at solid/water interfaces.

Transformation of a 2D gas layer into an INB was also reported in a TEM study of growth of hydrogen INBs^40^ as well as in an AFM study at HOPG/water interface^41^. Both observations showed initial shrinking of the lateral size of the 2D layers followed by gradual increase in height, consistent with our observations.

### Interfacial gas hydrate phases

We suspect that the fluid structures observed here are aggregates of pure gas molecules (nitrogen and oxygen) confined at the HOPG/water interface, where there are only weak van der Waals interactions among these gas molecules. Our observations indicate that ordered domains appear only in the areas outside or at the perimeter of the fluid regions (Figs 1, 2, 4 and 5 as well as Supplementary Figs S2, S3 and S5), suggesting that the presence of water is essential for formation of these ordered structures. The rather slow nucleation and growth processes of the ordered domains relative to the appearance of the fluid layers in water (Figs 1, 2 and 4 as well as Supplementary Figs S2 and S3) suggests that the formation of ordered domains requires a complicated bonding arrangement of water and gas molecules, rather than a simple aggregation of pure gas molecules at the interface. Thus, the gas-containing ordered structures detected here bear some resemblance to bulk gas hydrates in which water molecules are linked through hydrogen bonding and create cavities that harbour gas molecules^42^,^43^.

Hydrogen-bonded networks of water can stabilise gas-containing ordered structures at the interface, explaining why the ordered structures exhibit mechanical properties that are very different from those of the fluid structures (Figs 2 and 4 as well as Supplementary Figs S2 and S3). Interfacial gas-hydrate phases would be more hydrophilic than bare HOPG surface, resulting in less adhesion and dissipation on AFM (Figs 2 and 4 as well as Supplementary Figs S2 and S3). Hydrophilic gas-hydrate domains act as barriers to the lateral growth of fluid structures, which preferentially adsorb onto bare HOPG regions (hydrophobic regions) in water. Similarly, the step edges of the HOPG substrate confined the lateral growth of the fluid layers, probably because they are hydrophilic due to broken bonds. These hydrophilic structures may serve as pinning sites for the lateral confinement of the fluid layers and INBs. Pinning of the three-phase INB-water-surface contact line was previously attributed to omnipresent chemical and geometrical surface heterogeneities^21^. Such surface heterogeneities were also considered to underlie contact angle hysteresis^22^,^23^,^24^. Our observations of substrate step edges and the formation of ordered domains provide microscopic evidence of the origin of such surface heterogeneities. This evidence is remarkable, because we began our experiments with a relatively homogeneous and clean HOPG surface in pure water (see Methods). Gases dissolved in water segregate to the interface, rendering the interface highly heterogeneous on the nanometre scale. Therefore, future experimental studies (with probe size >100 nm) and theoretical modelling of the interface will need to consider the heterogeneous nature of the hydrophobic/water interface demonstrated here.

We have demonstrated that the ordered domains play a crucial role in the formation of INBs. Since the presence of INBs has been reported for many other hydrophobic/water interfaces, the phenomena reported here may also occur at those interfaces. It will therefore be interesting to determine whether these hydrophilic domains also appear at other solid/water interfaces. Future investigations should address important issues related to the ordered structures and the interfacial gas hydrate phases, such as details of their molecular configurations, their chemical compositions, their phase diagrams, and their effects on electrochemical reactions and many other processes at hydrophobic/water interfaces. These subjects are fundamentally important and will underlie better understanding of and control over hydrophobic/water interfaces.

It is typically understood that heterogeneous nucleation of a new phase (or a new structure) occurs more easily than homogeneous nucleation due to reduction in the nucleation barrier height. Formation of interfacial nitrogen or oxygen hydrates at the HOPG/water interface under ambient conditions implies that gas hydrates may be induced or catalyzed at certain solid/water interfaces. This concept is consistent with a recent report that the nucleation and growth of methane hydrates in the confined nanospace of activated carbons occur under milder conditions with faster kinetics than nature^44^. Thus the HOPG/water interface may provide a good model system to study this fundamentally and technologically important phenomenon.

Formation of the gas-containing ordered structures occurs at the HOPG/water even when undersaturated water is used. This would have great implications on electrochemical reactions using HOPG, graphene, or other sp^2^ carbon materials as the electrodes. A recent study indicates that the history of the HOPG surface strongly influences the electrochemical behavior^45^. Initial cyclic voltammetry (CV) is close to reversible on freshly cleaved HOPG surfaces, but markedly diminished electron transfer (ET) kinetics was seen with extended exposure of the HOPG surface to repeated CV measurements. The deterioration in apparent ET kinetics was attributed to deposition of material on the HOPG electrode. Our observation of gradual formation of nitrogen and oxygen-containing ordered domains provides explanation of the phenomenon. It would also be interesting to explore whether other hydrophobic solids also exhibit interfacial gas hydrate phases in water.

### Model for the formation of gas structures at the HOPG/water interface

**Figure 6:**
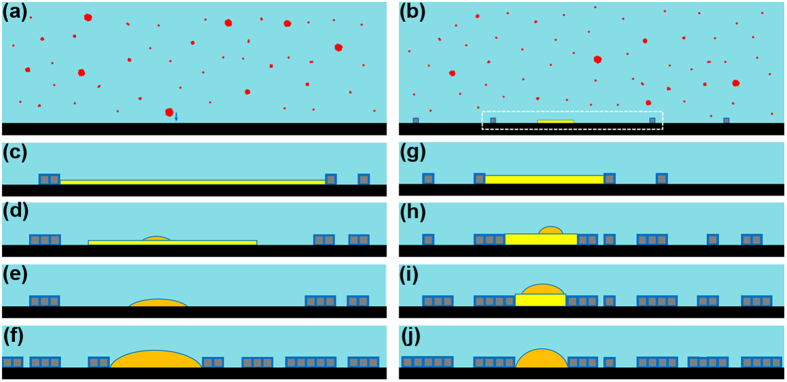
Scenario for the formation of gas structures at the HOPG/water interface. (**a**) Dissolved gas molecules (red) are present in clusters of various sizes. (**b**) Adsorption of a large gas cluster leads to the formation of a circular fluid layer (yellow). Ordered domains (grey) form through the adsorption of monomers and bonding with interfacial water molecules. Subsequent evolution is focused on the solid/water interface (white dashed box). (**c**–**f**) The case of a large confined fluid region and formation of an INB (orange). (**g**–**j**) The case of a small confined fluid region and formation of an INB. For clarity, some structures are not represented to scale.

Figure 6 depicts a scenario to explain our AFM-based observations. In gas-supersaturated water, dissolved gas molecules form clusters of various sizes (Fig. 6a; Supplementary Note 4). Adsorption of clusters larger than a certain size (which remains to be determined) leads to the formation of a circular fluid layer one molecule in thickness at the hydrophobic/water interface (Fig. 6b). Adsorption of monomers or small clusters of dissolved gas molecules outside the fluid regions and their bonding arrangement with the interfacial water molecules leads to the gradual nucleation and growth of ordered domains. The fluid layer increases in diameter through further adsorption of gas molecules. The lateral spreading tendency of the fluid layer is hindered when its edge contacts immobile hydrophilic defects, such as substrate step edges or ordered domains. The ordered domains increase in coverage over time and eventually confine the fluid layer within a certain region; the ordered domains may also nucleate at the perimeter of the fluid layer. The subsequent development of the confined fluid region varies with its lateral size. Figure 6c–f illustrate a transformation process in a large confined area. Further adsorption of a certain number of gas molecules onto a large confined fluid regions leads to instability of the 2D layer, which is subsequently transformed into a cap-shaped INB of a smaller diameter through shrinking of the fluid layer (Fig. 6d,e). The ordered domains gradually nucleate in the region originally covered by the fluid layer and eventually confine the INB within a small region (Fig. 6f).

Figure 6g–j illustrate another scenario in which the diameter of the confined region is small. Incoming gas molecules increase the thickness of the 2D layer (Fig. 6g). In addition, the edge of the fluid layer can be gradually converted into ordered domains through rearrangement of the gas molecules at the perimeter of the fluid layers with the surrounding water, gradually decreasing the lateral area of the confined region (Fig. 6h,i). At a certain point, a cap-shaped structure appears on the 2D layer (Fig. 6h). The cap-shaped structure initially grows in both the lateral and vertical dimensions (Fig. 6h,i). When its lateral size reaches the edge of the confined region, further gas adsorption leads to an increase in height only (Fig. 6j).

Here, 2D circular layers often appeared near substrate step edges or other line defects of the HOPG substrate (Figs 1 and 2 as well as Supplementary Figs S3 and S5), suggesting that the initial fluid layers may be mobile at the interface and tend to persist near these line defects. We expect the mobility of a fluid layer to increase as its lateral size decreases. The irregular region indicated with a blue arrow in Fig. 2 may be due to a small fluid layer that moves rapidly around the sparsely scattered ordered domains. A larger fluid layer may be less mobile and more easily confined by scattered ordered domains.

### Nucleation of INBs and gas-supersaturated state

INBs are generally prepared using gas-supersaturated water^1^,^6^,^7^,^8^,^9^. Interestingly, we also detected the formation of INBs after we deposited water that was rapidly heated to 45 °C (Fig. 2). Because the water cools to the temperature of the substrate (room temperature) within 10 s of deposition^46^, it becomes undersaturated (60% of the saturation concentration at 25 °C; Methods) soon after deposition. In this investigation, we conducted seven independent experiments with rapidly heated water (one example is shown in Fig. 2); INBs were evident in all of them (data not shown). In comparison, deionised water taken directly from water purifiers typically contains oxygen at 60–80% of the saturation concentration at 25 °C. We conducted tens of experiments using fresh deionised water, yet we only observed ordered domains—never the formation of INBs (data not shown). Our observations are therefore consistent with previous reports that no INBs are observed at the HOPG/water interface when deionised water is deposited^47^,^48^, that the density of INBs increases with increasing water temperature^46^,^49^,^50^, and that gas supersaturation is not required for the nucleation of INBs^46^.

We were also interested to note that INBs continued to grow in height even in undersaturated water (Fig. 2). This continuous increase over several hours to one day also occurred in many other cases, including after the deposition of chilled water (Figs 4 and 5) and after the deposition of rapidly heated water (Fig. 2). These observations reflect the strong tendency for dissolved gas molecules to adsorb at hydrophobic/water interfaces, considering that the dissolved gas concentration should decrease over time due to gas adsorption.

Our observations clearly indicate that gas concentration alone does not govern the behaviours of gases dissolved in water and gas accumulation at solid/water interfaces. For each gas concentration, there may be a cluster size distribution that is in thermodynamic equilibrium. A higher gas concentration would have many large clusters. A sudden increase in temperature likely causes the rapid aggregation of dissolved gas molecules into larger clusters (a supersaturated state); the concentration of large clusters increases at the expense of smaller clusters or monomers. Some large clusters are transformed into gas bubbles at the wall of the water container during heating, leading to the removal of a certain amount of gas from water (a reduction in the dissolved gas concentration). However, some large gas clusters remain dissolved in water, and an extended period of time may be required for the gas clusters to relax back to thermodynamic equilibrium after the water cools to room temperature. This scenario explains the formation of INBs using rapidly heated water and the lack of INB formation after the deposition of fresh deionised water, even though the gas concentrations in both cases are similar. Since decompression also leads to the formation of gas bubbles microns in size or larger, similar to the case of a sudden increase in water temperature, we expect that a decrease in pressure may also cause dissolved gas molecules to aggregate into larger clusters. Thus, water may be in a gas-supersaturated state temporarily even though the gas concentration is smaller than the saturation level.

## Discussion

This work underlies the importance of microscopic observations of the formation processes of various gas-containing structures at solid/water interfaces using highly sensitive AFM. Our unprecedented observations of the details of INB formation reveal the states of gas at solid/water interfaces as well as their dynamic behaviors. The observations also indicate an intricate interplay among gas molecules, water, and hydrophobic solids, which provide important clues to understand microscopic interactions among gas molecules, water, and hydrophobic solids. Further theoretical and experimental investigations will lead to better understanding of many phenomena at hydrophobic/water interfaces.

## Methods

### Sample and water preparation

HOPG substrate (12 mm × 12 mm, ZYB grade from Momentive) was freshly cleaved (by peeling off the top layer with Scotch tape) to expose a clean and atomically flat surface. Water was purified with a Milli-Q system (Millipore Corp.) with a resistivity of 18.2 MΩ·cm. Slightly air-supersaturated water was prepared by storing purified Milli-Q water with air in a sealed 50-ml conical centrifuge tube at 4 °C in a refrigerator for several days. Chilled water was either (i) deposited on a HOPG substrate or (ii) heated to 45 °C in a 95 °C hot bath before water deposition (Supplementary Fig. S6). Water was extracted with a pipette and deposited onto the freshly cleaved HOPG substrate, which was kept at room temperature (23–25 °C) in a closed fluid cell equipped with AFM. The oxygen concentration of the chilled water (~4 °C) was measured as 11~12 mg/l using a dissolved oxygen tracer (Lamotte 1761M) (data not shown). When the chilled water was heated to 45 °C, the oxygen concentration was ~5 mg/l (data not shown), or ~60% of the saturation oxygen concentration at 25 °C (8.3 mg/l). Gas bubbles were evident at the inner wall of the centrifuge tube, explaining the decreased oxygen concentration in this rapidly heated water. The water volume in the AFM liquid cell was only 60 μl, and the water temperature was expected to stabilise near room temperature within 10 s after injection into the liquid cell.

### Atomic force microscopy

AFM was performed with a modified Bruker AXS Multimode NanoScope V at room temperature (23–25 °C). FM (Supplementary Fig. S7a) and PF (Supplementary Fig. S7b) modes were used. FM mode was used here because it achieves high force sensitivity in water and more accurately measures the surface profile of INBs and other fluid structures than other operation modes^29^,^51^; however, more time (10–20 min) is required to tune the imaging conditions before imaging can begin. In addition, stable operation in FM mode may not be achieved for certain tips. With PF mode, AFM can begin sooner (5–10 min) after water deposition and stable imaging can be achieved easily. However, care must be taken when interpreting topographic images acquired in PF mode^29^. We used Si cantilevers (OMCL-AC240TS from Olympus) with a spring constant of 0.7~3.8 N/m, a nominal tip radius of ~10 nm, and a free resonance frequency of ~30 kHz in water. In FM mode, the oscillation amplitude was maintained at 1.0–1.5 nm; the set point was 13–20 Hz.

## Additional Information

**How to cite this article**: Fang, C.-K. *et al*. Nucleation processes of nanobubbles at a solid/water interface. *Sci. Rep*. **6**, 24651; doi: 10.1038/srep24651 (2016).

## Supplementary Material

Supplementary Information
