# Supplementary material for: Nucleation processes of nanobubbles at a solid/water interface
Source: Sci Rep. 2016 Apr 19;6:24651. doi: 10.1038/srep24651 (PMC4835695; doi:10.1038/srep24651)
Supplement: Supplementary Information [file srep24651-s1.pdf]

Supplementary Information

Nucleation Processes of Nanobubbles at a Solid/Water Interface

Chung-Kai Fang, Hsien-Chen Ko, Chih-Wen Yang, Yi-Hsien Lu, and Ing-Shouh Hwang*

Contents:

1. Supplementary Notes

2. Supplementary Figures

3. Supplementary References

Supplementary Notes

1. Driving force for the spreading of the adsorbed gas film at the HOPG/water interface.

Spreading of the adsorbed gas film at the interface is likely driven by gas-graphite interactions, which are much larger than the gas-gas interactions in this system. For example, the energy values of N_2–N_2, N_2–HOPG, O_2–O_2, and O_2–HOPG interactions are approximately 9 meV, 100 meV, 10 meV, and 100 meV, respectively[1], suggesting that the stronger gas-HOPG interactions cause the adsorbed gas film to spread. However, the gas-HOPG interactions are unlikely to be very strong due to the van der Waals nature of the interaction. Thus, an unfavourable interaction (high interfacial tension) between the adsorbed gas structure and water reduces the spreading tendency of the structure, driving it to shrink in the lateral dimension and bulge in the vertical dimension to reduce the interfacial area. The formation of a gas film one molecule in thickness indicates that its interfacial tension with water should be much smaller than the surface tension of water. Interfacial water may form a favourable hydration structure with the smooth gas film. Adsorption of excess gas molecules on the smooth gas film may disrupt the favourable hydration structure and cause the interfacial tension to increase, which becomes the driving force for the transition to a cap-shaped structure at a later time.

2. Interfacial tension between the fluid structures and water.

Formation of a complete wetting film at the HOPG/water interface implies that the spreading coefficient[2] $S \equiv \sigma_{HOPG\text{-}water} - (\sigma_{HOPG\text{-}film} + \sigma_{film\text{-}water}) > 0$, where $\sigma_{HOPG\text{-}water}$, $\sigma_{HOPG\text{-}film}$, and $\sigma_{film\text{-}water}$ represent the interfacial tension of HOPG/water, gas-film/HOPG, and gas-film/water, respectively. As estimated below, $\sigma_{HOPG\text{-}water}$ is smaller than the surface tension of water ($\sigma_{air\text{-}water}$ = 72 mN/m). Although the value of $\sigma_{HOPG\text{-}film}$ is not known, it should be small due to the lack of strong bonding between gas molecules (nitrogen or oxygen) and the HOPG substrate. Thus $\sigma_{film\text{-}water} < \sigma_{HOPG\text{-}\text{-}water} < \sigma_{air\text{-}water}$.

According to Young's law, $\cos\theta_{eq} = (\sigma_{\alpha\gamma} - \sigma_{\beta\gamma})/\sigma_{\alpha\beta}$ (refer to the figure below).

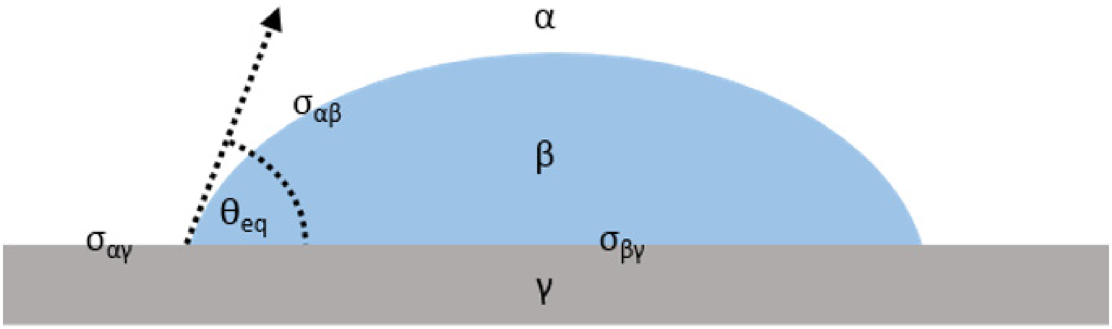

Since the contact angle of water on freshly cleaved HOPG is 65°[3], we estimate $\sigma_{HOPG\text{-}water}$ based on Young's law and the surface tensions of water and HOPG. The surface tension of water, $\sigma_{air\text{-}water}$, is 72 mN/m, and that of HOPG is 35-55 mN/m[4,5]. Thus, $\sigma_{HOPG\text{-}water}$ is estimated to be 25 mN/m or smaller.

For an interfacial nanobubble (INB) at a solid/water interface, the typical contact angle is 5°-25°. The interfacial tension between an INB and water, $\sigma_{INB\text{-}water}$, should be somewhat larger than $\sigma_{film\text{-}water}$, but still considerably smaller than the surface tension of water. The small interfacial tension would make the INB highly

deformable and tend to adopt a rather flat morphology, which is very different from the stiff semispherical shape typically seen for a gas bubble of micron size or larger at a solid/water interface. Small interfacial tension also contributes to lower free energies of INBs and explain the high stability of INBs.

3. Nanobubbles as the micronuclei for gas bubble formation in water

It has been theoretically predicted that a vapour cavity will form only when the liquid is under extremely large tension[6]. However, these large tensile strengths have never been observed, leading to the introduction of the idea of micronuclei. Plesset stated in 1969[7] that small gas bubbles cannot be the persistent nuclei for the formation of micro- or macro-bubbles in water due to their short lifetimes[6]. Plesset also conjectured[7] that dissolved gases may form adsorbed films of gas on hydrophobic surfaces; these films would have a low surface tension and could act as nucleation centres for bubble formation in water. Arieli and Marmur[8] proposed that surface nanobubbles may be the gas micronuclei responsible for the bubbles that cause decompression sickness, but the mechanism was not known. Our study indicates that INBs are gas aggregates (or gas condensates) with a low interfacial tension with water, providing insight into the nucleation of gas bubbles in water.

4. Gas cluster distribution in water.

The two types of gas-containing interfacial structures observed in the present investigation, ordered domains and fluid structures, may originate from different gas configurations in bulk water. During numerous atomic force microscopy (AFM) studies of the HOPG/water interface that we conducted under ambient conditions1,[9-]

[12], we typically observed INBs or fluid layers when air-saturated or gas-supersaturated water was deposited. Ordered domains appeared in saturated or super-saturated water as well as in partially degassed water[9]. In partially degassed water, it is expected that dissolved gas molecules are present mainly in the form of well-dispersed monomers; thus, ordered domains form through self-assembly of individual monomers or small clusters at the interface. In air-supersaturated water, a subset of the dissolved gas molecules probably aggregates into large clusters. Theoretical studies have indicated that small non-polar molecules can aggregate into clusters nanometres in size in bulk water[13,14]. Nitrogen and oxygen, the two major components of air, are non-polar, suggesting that they may aggregate into large clusters with a diameter >1 nm when the gas concentration is near or above the saturation level. Adsorption of a large cluster at the HOPG/water interface may lead to the formation of a circular fluid layer that later evolves into a cap-shaped INB.

Supplementary Figures

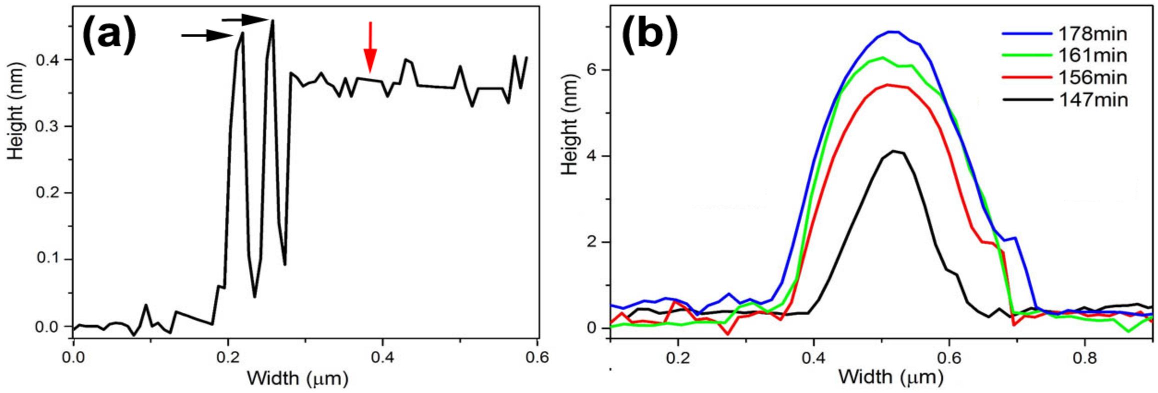

Figure S1 Height profiles measured in Figure 1 of the main text. (a) Height profiles along the dashed line in Figure 1b of the main text. Black arrows indicate the profile across ordered domains; the red arrow indicates the profile across a fluid layer. (b) Evolution of the height profiles of the INB in region 4 at different times.

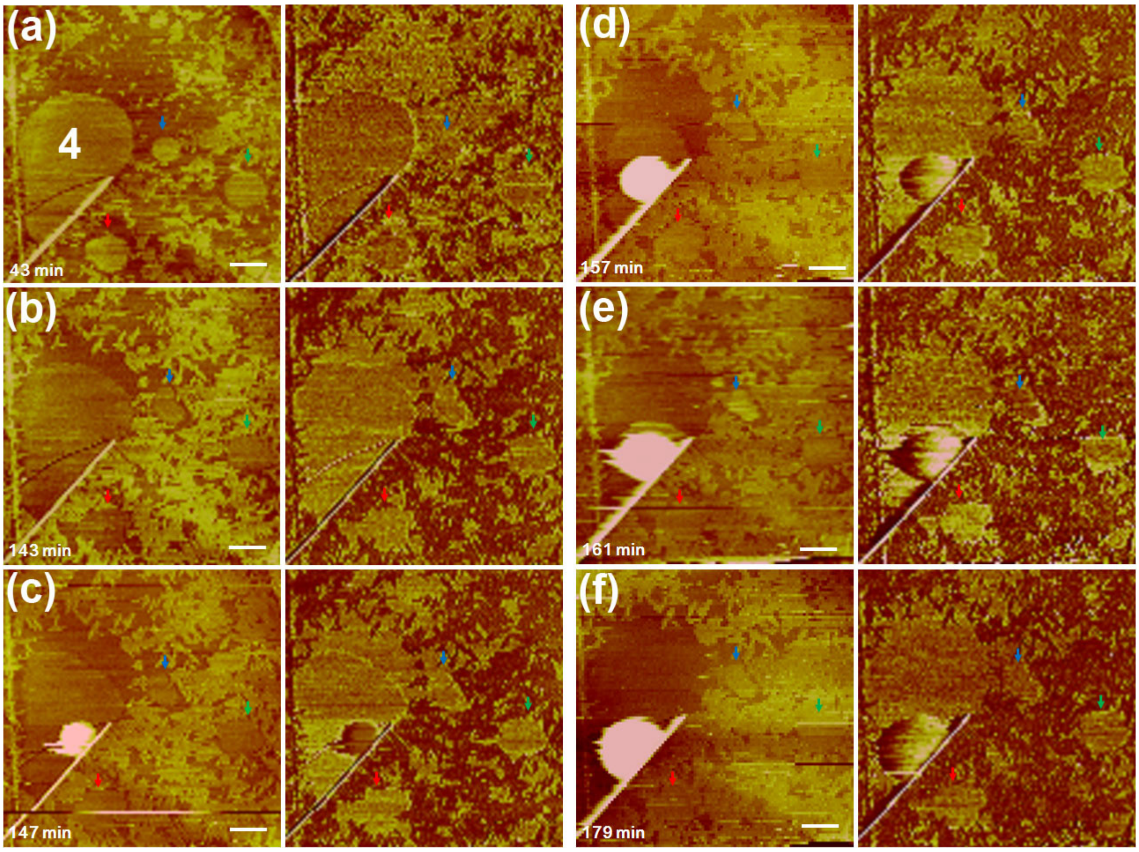

Figure S2 AFM images of the transformation process around region 4 in Figure 1 of the main text. The left and right columns of each panel depict the topographic and dissipation images, respectively. Three small fluid layers are indicated with coloured arrows. The fluid layers exhibited a lower height than the ordered domains in **(a-c)**, but they grew in height at later stages and could not be distinguished easily from the surrounding ordered domains in topographic images. The ordered domains displayed a lower dissipation than the fluid layers and bare HOPG regions; thus, the dissipation images enabled us to better distinguish the ordered domains from other regions. Scale bar, 200 nm.

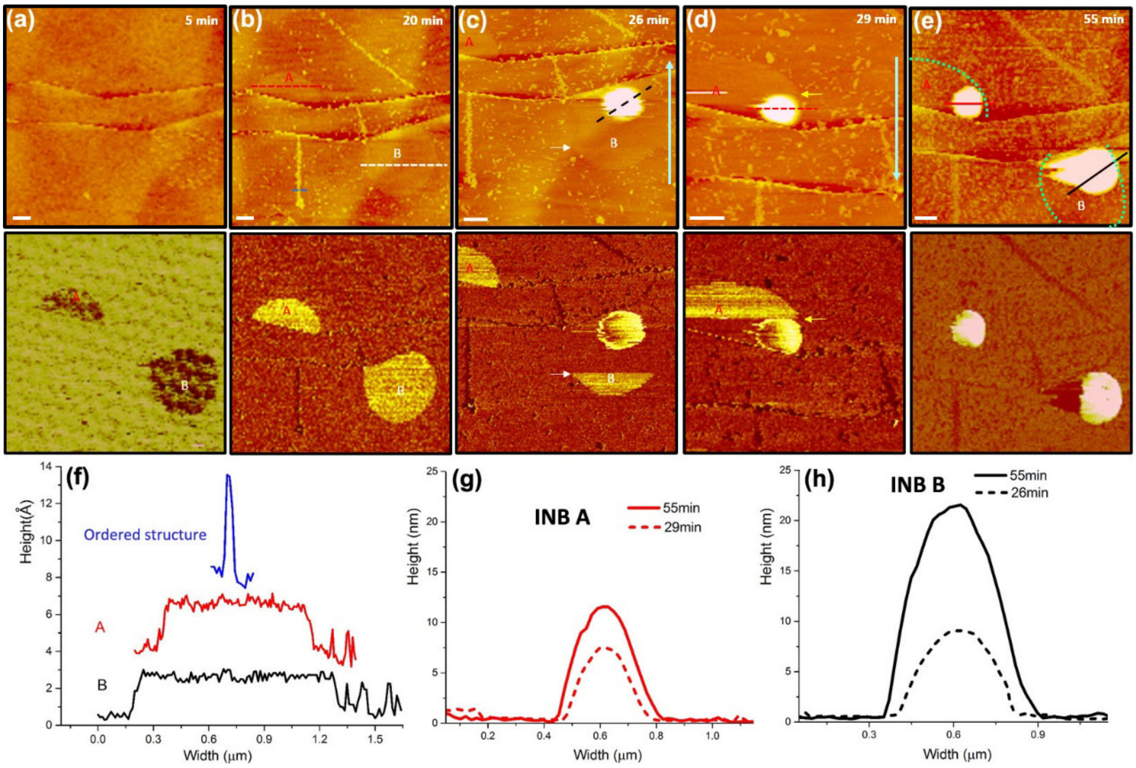

Figure S3 AFM of the transition of large fluid layers to INBs at a HOPG/water interface. (a) Topographic (top) and adhesion (bottom) images acquired in PF mode at t=5 min. Scale bar in all panels, 250 nm. The adhesion map provides considerably higher contrast for the two circular layers (indicated with A and B) than does the topographic map. Adsorption of circular layers had already occurred at t=5 min. Imaging was subsequently switched to FM mode; **(b)-(e)** contain the topographic (top) and dissipation (bottom) images acquired with this mode at various times. The topographic images also show two circular layers ~0.3 nm in thickness, and the dissipation maps exhibit strong contrast for the fluid regions relative to other parts of the interface. The small bright speckles in the topographic images are ordered domains; they appear darker than the bare HOPG surface in the dissipation images. In **(c)**, circular layer B disappears suddenly at the scan line indicated by a white arrow, and a cap-shaped nanostructure appears at subsequent scan lines. Note that the images were acquired through raster scanning; blue arrows at the side of the topographic images

indicate the slow scan direction. Each frame took ~3 min to acquire. The time to scan one line was 1 s. In **(d)**, circular layer A suddenly transforms into a cap-shaped nanostructure. In **(e)**, the two cap-shaped INBs grow larger in both the vertical and lateral dimensions. In addition, small ordered domains (bright speckles) appear inside the regions that were originally covered by the thin layers (outlined by green dashed lines). **(f)** Height profiles in **(b)**. **(g)** Height profile across INB A at two time points. **(h)** Height profile across INB B at two time points. Both INBs exhibited growth in the vertical and lateral directions.

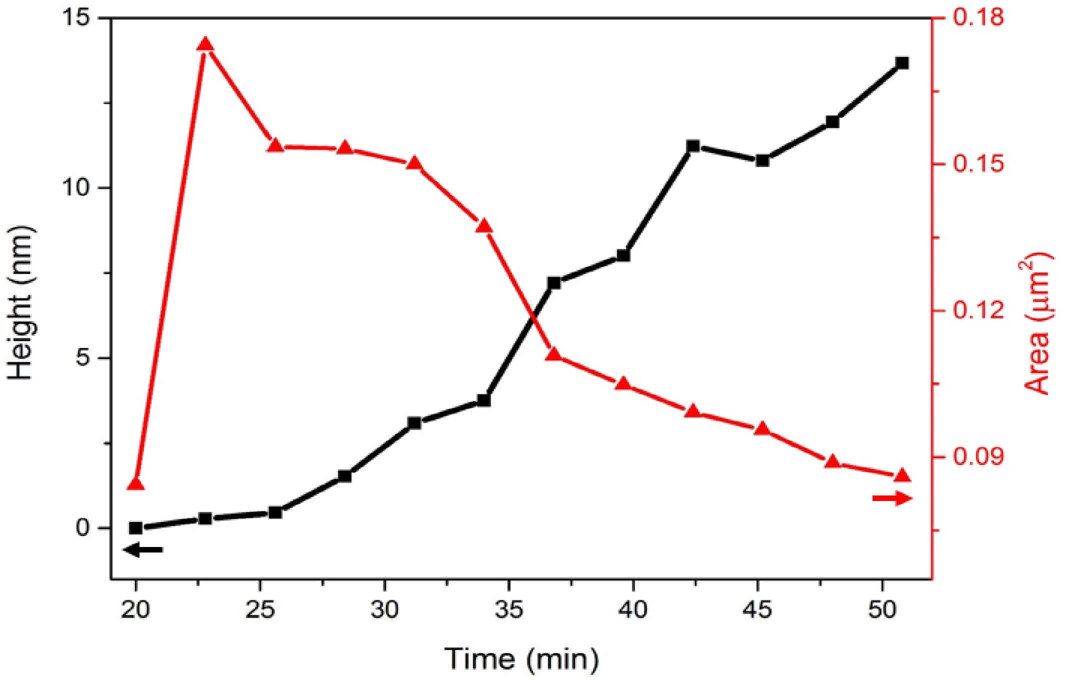

Figure S4 Evolution of lateral area and apparent height over time for the fluid structure indicated with a white arrow in Figure 2 of the main text. Lateral area was measured from the stiffness maps and apparent height was measured from the highest point of the height profiles.

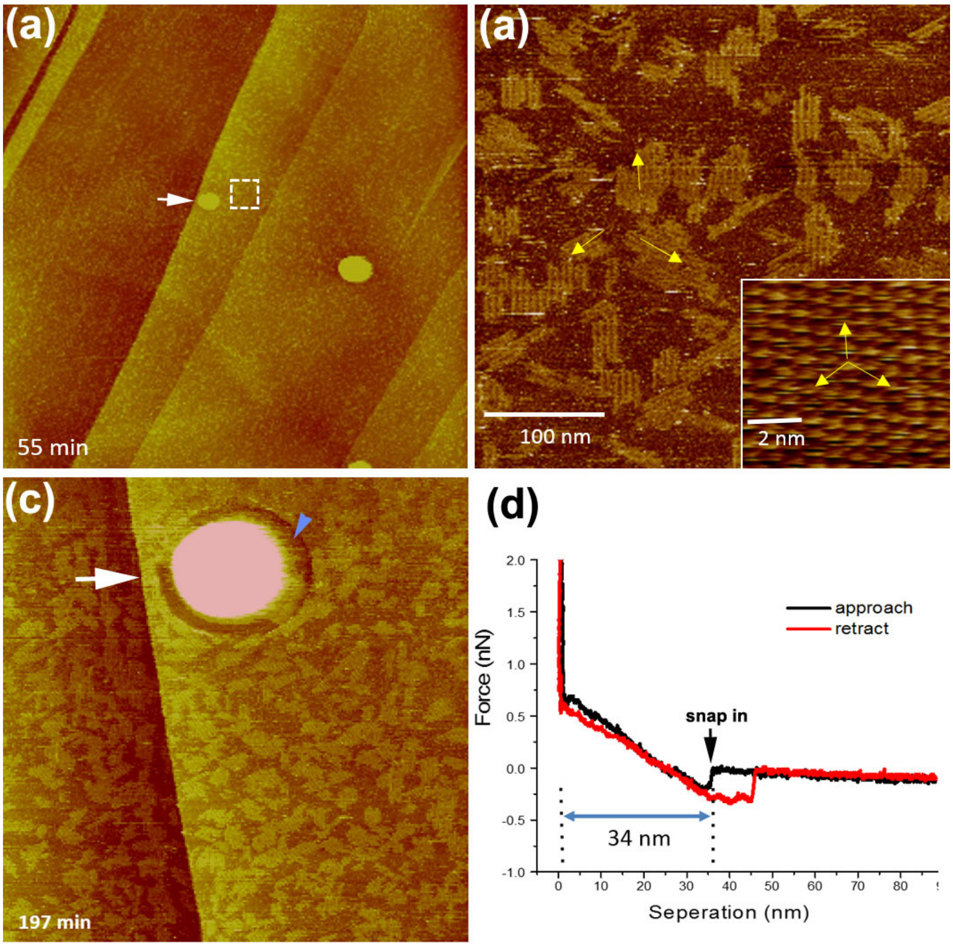

Figure S5 Topographic (PF, 250 pN) images of structures at the HOPG-water interface acquired at *t*= 55 min (a) and *t*= 71 min (b), and t= 197 min (c). The INB indicated with a white arrow serves as a marker for comparison with images shown in Figure 2 of the main text. Two additional INBs were observed outside the scan area shown in Figure 2 of the main text. The image shown in **(b)** was acquired in the outlined region in **(a)**. It shows that the white speckles in (**a**) are small ordered domains. The yellow arrows in **(b)** indicate the three orientations of the row-like structures, which are parallel with the lattice orientations of the HOPG substrate, zig-zag directions. The HOPG lattice was acquired at a high loading force with the contact mode (inset). The images in (**b**) and (**c**) were acquired with the fast scan direction rotated 30° counter-

clockwise relative to that in (**a**). The image size is 5 μm × 5 μm (**a**) and 1 μm× 1 μm (**c**). (**d**) Force-distance curve measured on the INB. When the tip approached the INB, snap-in occurred when the tip touched the INB. A positive peak force was required for stable AFM imaging of the entire surface; thus, the tip needed to pierce the INB to a certain depth to offset the attractive snap-in force. This results in a dark ring, indicated with a blue arrow in (**c**), at the outer region the INB. In the dark ring region, the tip penetrated through the thin fluid region to reveal the HOPG substrate. Surrounding the INB, ordered domains were present and they might be responsible for the confinement of the fluid region and the observed pinning of the three-phase contact line in Fig. 3b.

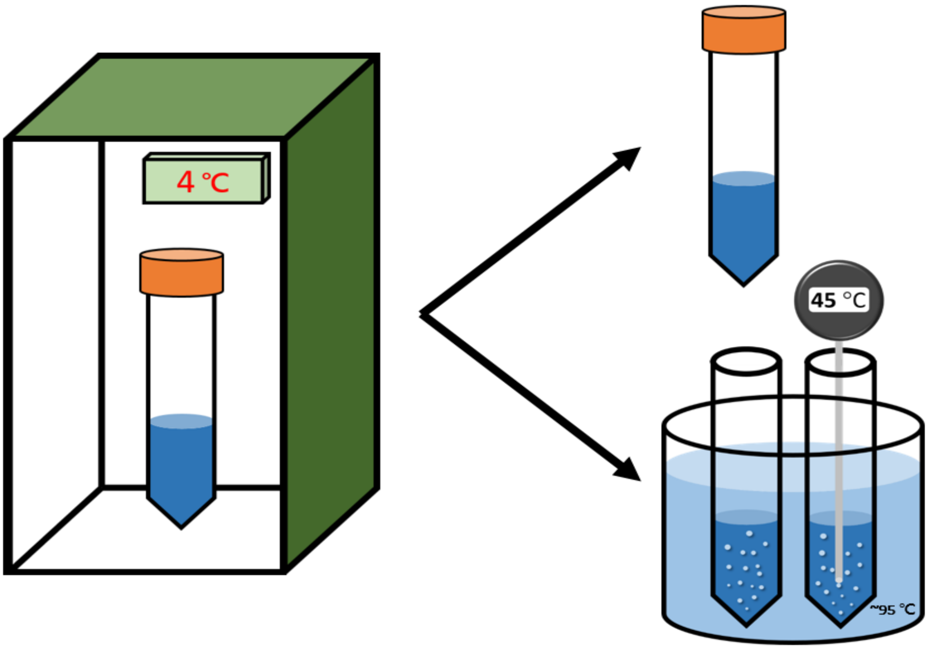

Figure S6 Water preparation before AFM. Purified deionized water was first stored with air in a sealed centrifuge tube at 4°C in a refrigerator for several days. The chilled water could be either (i) deposited on a HOPG substrate in a AFM liquid cell at room temperature or (ii) heated to 45°C in a 95°C hot water bath before deposition on a HOPG substrate in a liquid cell.

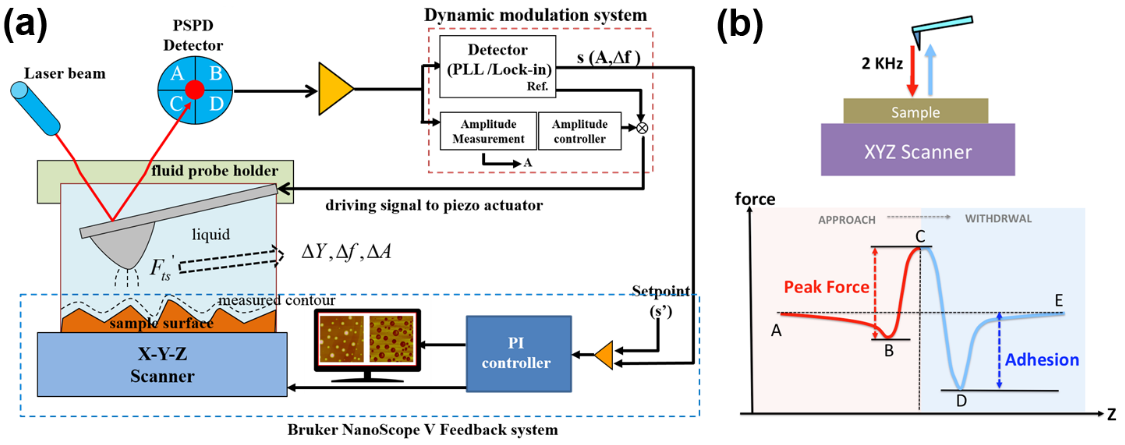

Figure S7 AFM operation. (a) Schematic of the Multimode NanoScope V modified for FM mode. Oscillation of the cantilever was driven using a dynamic-modulation system comprised of a lock-in unit/phase-lock-loop (PLL) unit (Nanonis OC4 Station from SPECS) and a signal-access module (Bruker AXS). The PLL unit was employed to track the resonance frequency of the vibrating cantilever. The resonance frequency shift (Δf) was used as the feedback input signal of a proportional-integral controller to obtain topographic images. The driving amplitude, which we controlled with the PLL in order to maintain a constant amplitude of cantilever oscillation during scanning, was measured and recorded simultaneously. The amplitude value was typically considered to be the energy dissipation signal[15]. A dissipation map was acquired along with the topographic images. **(b)** Illustrations of PF mode. Vertical piezo movement results in cycles of approaching and retracting traces in which the tip makes intermittent contact with the sample surface, yielding force-distance curves. Topography information is obtained from the height correction performed by the feedback loop to keep a constant “peak” of force, while the slope of the contact region determines the stiffness of the sample at each pixel. Adhesion and deformation can also be extracted from the force curves. Our previous studies indicated that snap-in usually occurs when the AFM tip touches a fluid region such as an INB. The tip must penetrate the structures to a certain

depth to offset the attractive snap-in force to reach a positive preset peak force[1]; thus, the penetration depth, which varied with the hydrophobicity of the AFM tip[16], should be added to the height profile of the fluid region obtained from the topography images obtained in PF mode. If a fluid structure is thinner than the penetration depth, it cannot be seen in the topographic images because the tip traces the profile of the stiff structure underneath. Snap-in, which is caused by the hydrophobicity of the tip apex and the fluid nature of the structure under study[16], does not strongly affect topographic images acquired in FM mode[1], probably because the feedback signal (the resonance frequency shift) is related to the force gradient rather than to the interaction force itself. A sharp increase in the resonance frequency often occurs when the tip touches a fluid structure[1].

Supplementary References

1. Lu, Y. H., Yang, C. W., Fang, C. K., Ko, H. C. & Hwang, I. S. Interface-induced ordering of gas molecules confined in a small space. *Sci. Rep*. 4, 7189 (2014).
2. de Gennes, P. G. Wetting: statics and dynamics. *Rev. Mod. Phys.* **57,** 827-863 (1985).
3. Li, Z. *et al.* Effect of airborne contaminants on the wettability of supported graphene and graphite. *Nature Materials* **12**, 925–931 (2013).
4. Morcos, I. Surface tension of stress-annealed pyrolytic graphite, *J. Chem. Phys.* **57**, 1801– 1802 (1972).
5. Wang, S., Zhang, Y., Abidi, N. & Cabrales, L. Wettability and Surface Free Energy of Graphene Films. *Langmuir* **25**, 11078-11081 (2009).
6. Plesset, Milton S. *Tensile Strength of Liquids.* Rep. 85-46, Calif. Inst. of Tech (1969).
7. Epstein, P. S. & Plesset, M. S. On the stability of gas bubbles in liquid-gas solutions. *J. Chem. Phys.* **18,** 1505-1509 (1950).
8. Arieli, R. & Marmur, A. Decompression sickness bubbles: are gas micronuclei formed on a flat hydrophobic surface?. *Respiratory Physiology & Neurobiology* **177,** 19-23 (2011).
9. Lu, Y. H., Yang, C. W. & Hwang, I. S. Molecular layer of gaslike domains at a hydrophobic-water interface observed by frequency-modulation atomic force microscopy. *Langmuir* **28,** 12691–12695 (2012).
10. Hwang, I. S., Yang, C. W. & Lu, Y. H. Evidence of epitaxial growth of molecular layers of dissolved gas at a hydrophobic/water interface. *arXiv* 1203.6696 (2012).
11. Yang, C.W., Lu, Y. H. & Hwang, I. S. Condensation of dissolved gas molecules at a hydrophobic/water interface. *Chin. J. Phys.* **51,** 250–262 (2013).
12. Lu, Y. H., Yang, C. W. & Hwang, I. S. Atomic Force Microscopy Study of Nitrogen Molecule Self-Assembly at the HOPG-Water Interface, *Appl. Surf. Sci.* **304**, 56-64 (2014).

13. Raschke, T. N., Tsai, J. & Levitt, M. Quantification of the hydrophobic interaction by simulations of the aggregation of small hydrophobic solutes in water. *Proc. Natl. Acad. Sci. USA* **98,** 5965–5969 (2001).
14. Chadler, D. Interfaces and the driving force of hydrophobic assembly. *Nature* **437,** 640-647 (2005).
15. Giessibl, F. J. Advance in atomic force microscopy. *Rev. Mod. Phys.* **75,** 949-983 (2003).
16. Song, Y., et al. The origin of the "snap-in" in the force curve between AFM probe and the water/gas interface of nanobubbles. *ChemPhysChem* **15,** 492-499 (2014).
